# Conception, synthesis, and characterization of a rofecoxib-combretastatin hybrid drug with potent cyclooxygenase-2 (COX-2) inhibiting and microtubule disrupting activities in colon cancer cell culture and xenograft models

**DOI:** 10.18632/oncotarget.25450

**Published:** 2018-05-25

**Authors:** Surendra R. Punganuru, Hanumantha Rao Madala, Constantinos M. Mikelis, Anshuman Dixit, Viswanath Arutla, Kalkunte S. Srivenugopal

**Affiliations:** ^1^ Department of Pharmaceutical Sciences, School of Pharmacy, Texas Tech University Health Sciences Center, Amarillo, TX, USA; ^2^ Institute of Life Sciences, Nalco Square, Bhubaneswar, Odisha, India

**Keywords:** COX-2, hybrid drugs, microtubule inhibitors, colon cancer, angiogenesis inhibitors

## Abstract

Tumor heterogeneity and drug resistance pose severe limitations to chemotherapy of colorectal cancers (CRCs) necessitating innovative approaches to trigger multiple cytocidal events for increased efficacy. Here, we developed a hybrid drug called KSS19 by combining the COX-2 selective NSAID rofecoxib with the cis-stilbene found in combretastatin A4 (CA4), a problematic, but potent antimicrotubule and anti-angiogenesis agent. The structural design of KSS19 completely prevented the isomerization of CA4 its biologically inactive trans-form. Molecular modeling showed that KSS19 bound avidly to the COX-2 active site and colchicine –binding site of tubulin, with similar docking scores of rofecoxib and CA4 respectively. KSS-19 showed potent anti-proliferative activity against a panel of colon cancer cell lines; HT29 cells, which are resistant to CA4 were 100 times more sensitive to KSS19. The hybrid drug potently inhibited the tubulin polymerization *in vitro* and in cells inducing a G2/M arrest and aberrant mitotic spindles. Both the basal and LPS-activated levels of COX-2 in colon cancer cells were highly suppressed by the KSS-19. The cancer cell migration/invasion was inhibited and accompanied by increased E-cadherin levels and activated NF-kB/Snail pathways in KSS19-treated cells. The drug also curtailed the formation of endothelial tubes in three-dimensional cultures of the HUVE cells at 250 nM, indicating strong anti-angiogenic properties. In subcutaneous HT29 colon cancer xenografts, KSS19, as a single agent (25 mg/kg/day) significantly inhibited the tumor growth and downregulated the intratumoral COX-2, Ki-67, the angiogenesis marker CD31, however, the cleaved caspase-3 was elevated. Collectively, KSS19 represents a rational hybrid drug with clinical relevance to CRC.

## INTRODUCTION

Colorectal cancer (CRC) is ranked as the third most common form of cancer worldwide [1] and the third and second most leading cause of cancer-related deaths in women and men respectively in the United States [2]. Clinical efficacy of current anticancer drugs against the CRC is hampered by various factors, including multidrug resistance (MDR) [3], and inter and intratumoral heterogeneity at the genotypic and molecular target levels besides pharmacokinetic considerations [4]. Therefore, there is an urgent need for the discovery of new alternative drugs with more efficient therapeutic impact on CRC. A recent new strategy for cancer treatment involves the design and synthesis of hybrid drugs, which comprise the incorporation of two drug pharmacophores in a single molecule with an intention to exert dual drug action [5]. Hybrid compounds affecting more than a single target have been considered as more efficient and potent anticancer agents since it is almost impossible to destroy the malignancies focusing on a single target [6].

Microtubules are highly dynamic cytoskeletal filaments consisting of αβ-tubulin heterodimers that play vital roles in a variety of cellular processes, including intracellular transport, cell motility, and proliferation [7, 8]. As tubulins play a central role in the duplication and segregation of chromosomes during mitosis, disruption of microtubule dynamics induces cell cycle arrest at the G2/M phase and activates signals for apoptosis [9]. Microtubule dynamics targeting ligands can be broadly divided into two categories; those that inhibit the formation of the mitotic spindle such as the colchicine and vinblastine and those that inhibit the disassembly of the mitotic spindle once it has formed, such as paclitaxel and docetaxel [10]. The three characterized binding sites of tubulin are the taxane domain, the vinca domain, and the colchicine domain, and most antimicrotubule agents interact with tubulins at these known sites [11]. Although several microtubule-targeting drugs are in clinical use, there remains a need to identify novel agents that can overcome the limitations of current therapies, including acquired and innate drug resistance and undesired adverse effects [12]. To overcome tumor resistance, much research has been directed at developing ligands that bind to colchicine domain [13, 14]. Several agents binding to the colchicine site have been identified as potential anticancer agents and their ability to overcome Pgp/β-III tubulin mediated drug resistance along with their antiangiogenic or antivascular actions have attracted much interest. Most of these compounds have small molecular weights with chemically modifiable functional groups; therefore they are amenable to chemical modification and improvement of pharmacokinetic (PK) properties, efficacy, and reduced toxicity.

Despite its simple molecular structure, Combretastatin A4 (CA4) is one of the most powerful inhibitors of tubulin polymerization through binding to the colchicine domain of tubulin [15]. CA4 and its water-soluble prodrug CA4P exhibit potent anti-proliferative activities against a wide spectrum of cancer cells including drug-resistant variants [16]. However, the drug is poorly soluble and can induce serious adverse effects. Further, the cis configuration of CA4 is prone to isomerize to the thermodynamically more stable trans-form during storage and administration, producing a dramatic reduction in both anti-tubulin and antiproliferative activities [17]. Furthermore, COX-2 overexpressing adenocarcinomas exemplified by HT29 cells are inherently resistant to CA4 and its synthetic derivatives through unknown mechanisms. Nevertheless, overexpression of P-glycoprotein (P-gp) encoded by the MDR1 gene, is a major impediment to successful chemotherapy for colorectal cancer [18]. A recent study provided the first direct evidence that COX-2 contributes to P-gp-mediated multidrug resistance through phosphorylation of c-Jun at Ser63/73 in colorectal cancer cells [19].

Cyclooxygenases (COXs) are a family of enzymes, which catalyze the rate-limiting step of prostaglandin biosynthesis [20]. Cyclooxygenase-2 (COX-2), the inducible isoform of cyclooxygenase is considered to play an important role in colorectal carcinogenesis and is often upregulated in colon cancers [21]. COX-2 is an immediate-early response gene normally absent from most cells but is induced mainly at sites of inflammation in response to the pro-inflammatory cytokines such as IL-1α/β, IFN-γ, and TNF-α produced in RAS-mutated cells [22]. Elevated COX-2 is associated with tumorigenesis [23] in multiple ways such as resistance to apoptosis [24], increased migration and invasion [25], enhanced formation of endothelial tubes linked in turn to increased angiogenesis [26] and subversion of the immune system [27]. COX-2 expressing CRCs also display aggressive growth rates and curtailing the COX-2 with selective inhibitors like celecoxib has evoked much interest [28]. Therefore, efforts were made to engineer a hybrid drug targeting the COX-2 and microtubule dynamics in this study.

## RESULTS AND DISCUSSION

### Design and synthesis of the hybrid drug KSS19

**Figure 1:**
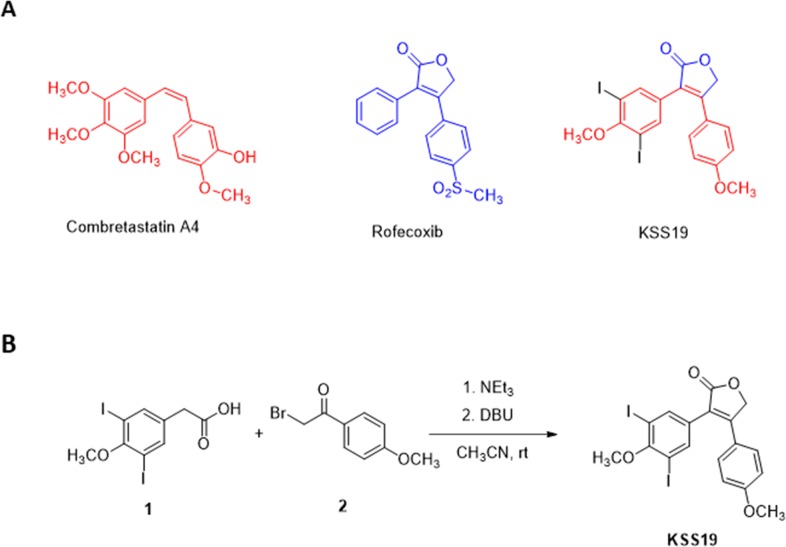
Chemical Structures of parent drugs and synthesis of KSS19 **(A)** Chemical structures of Combretastatin A4, Rofecoxib, and the hybrid compound KSS19. **(B)** Synthesis of KSS19 was achieved by reacting 2-(3,5-diiodo-4-methoxyphenyl) acetic acid and 2-bromo-1-(4-methoxyphenyl)ethan-1-one in the presence of a base using dichloromethane as solvent.

Although CA4 is a promising anti-mitotic clinical candidate, the metabolically unstable cis-olefinic bridge, drug resistance and toxicity to normal tissues hamper its clinical use. Therefore a number of studies have attempted to replace the olefinic bridge with more rigid and metabolically stable structures to maintain the correct conformation of the two adjacent phenyl groups [29]. Many of these new compounds have been shown to display an increased potency when compared to CA4 because of their increased stability. Higher expression of Cox-2, its role malignant progression and Cox-2 induced P-glycoprotein (P-gp), the product of the *MDR1* gene, are other prominent characteristics of CRC. As such, COX-2 inhibitors such as the celecoxib and rofecoxib have been investigated to arrest CRC proliferation and to increase the chemotherapeutic efficacy [30]. Rofecoxib, whose brand name is Vioxx was a widely NSAID and was withdrawn by the manufacturer in 2004. However, this does not preclude its use as an investigational cancer drug. Taking these points in to consideration, in our systematic effort to develop a novel multi-targeting agents from synthetic small molecules [31], in the present work, we aimed to address both stability and drug resistance glitches of CA4 by replacing the olefinic bridge with a structure that imparts COX-2 inhibiting property without affecting the tubulin interaction of the original drug. Accordingly, a novel class of compound KSS19 was synthesized based on the structures of CA4 and known COX-2 inhibitor rofecoxib (Figure 1). This compound showed properties similar to CA4 but have greater potency in inhibiting CA4 resistant COX-2 overexpressing colon tumor cell growth. Two of methoxy groups of the CA4 pharmacophore, were, however, replaced with iodine in the hybrid drug and named KSS19 (Figure 1). The structural design of KSS19 preserved the CA4 nucleus in the cis-configuration and the furan-one ring present in the place of olefin prevented its isomerization to the biologically inactive trans-form. KSS19 was prepared in two steps under one-pot operation by first reacting 2-(3,5-diiodo-4-methoxyphenyl)acetic acid **1** with 2-bromo-1-(4-methoxyphenyl)ethan-1-one **2** in the presence of base triethylamine, followed cyclization using diazabicyclo[5.4.0]-undec-7-ene.

### *In vitro* cytotoxicity

**Figure 2:**
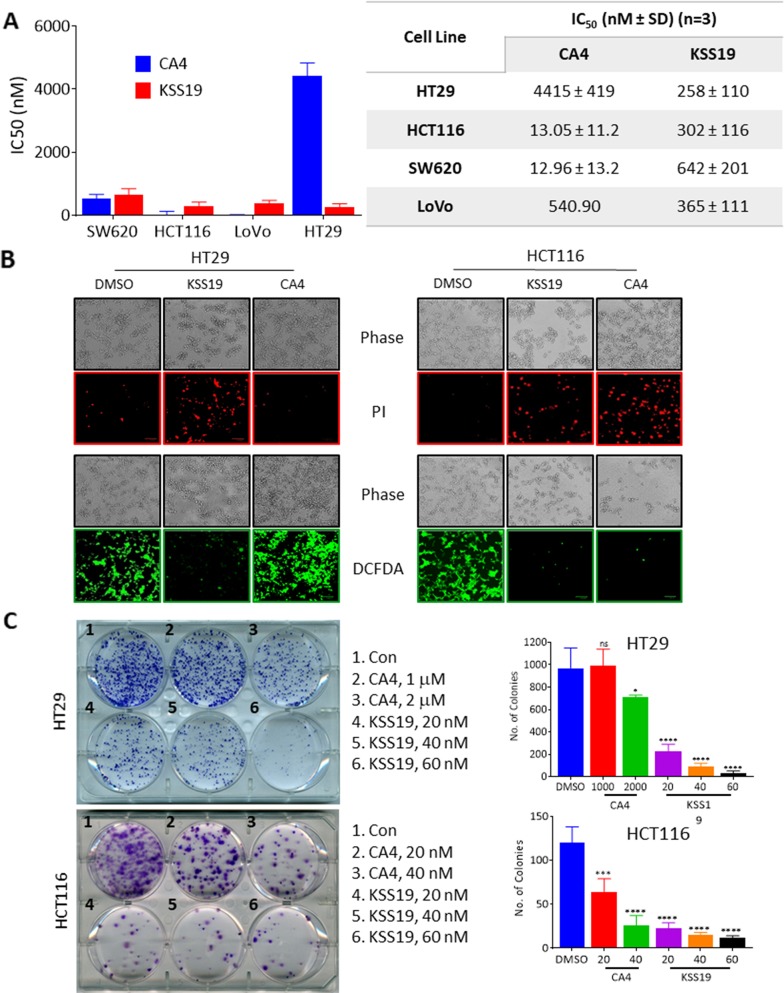
Cytotoxic effects of KSS19 on colon cancer cells **(A)** Colon cancer cells (HT29, HCT116, SW620, and LoVo) were treated with various concentrations of KSS19 or CA4 (0-50 μM) for 72 h, and the cell killing was determined by resazurin reduction assay. **(B)** Cell viability was assessed by fluorescence microscopy using DCFDA and PI staining. HT29 and HCT116 cells were treated with KSS19 and CA4 at their IC_50_ concentrations for 48 h, stained with DCFDA or PI and imaged using fluorescence microscopy. **(C)** Colony formation assays. HT29 and HCT116 cells were seeded in six-well plates at a density of 500 cells per well and the cells were treated with KSS19 and CA4 for 24h. Drug-containing media was replaced with fresh media and subsequently, cells were cultured for 21 days. In the end, cells were fixed and stained with crystal violet and images were photographed. Colony formation was quantified using ImageJ software. The bar graphs on right show the quantitation of cell colonies.

To explore the effect of KSS19 on CRC cell proliferation, we treated four human colon cancer cell lines (HT29, HCT116, SW620, LoVo) with KSS19 at increasing concentrations along with the parent drug CA4 as a control. Cell viability was measured using resazurin reduction assay [31]. Rofecoxib used as another control did not elicit significant cytotoxicity at a maximal concentration of 100 μM. However, the KSS19 was highly potent in curtailing the CRC proliferation in a concentration-dependent manner. The growth inhibition constants (IC_50_) of the different tumor cell lines ranged from 258 to 365 nM for KSS19 (Figure 2A). Interestingly, the HT29 cells, which are extremely resistant to CA4 were highly sensitive to (~17-fold decrease in the IC_50_) KSS19. While CA4 was relatively more cytotoxic to the other cell lines, KSS19 still strongly inhibited the cell growth at low submicromolar concentrations (Figure 2A). Next, the cytotoxic extent of KSS19 and CA4 against the HT29 and HCT116 cells was visualized by propidium iodide (PI) staining after 24 h drug treatment; the red nuclear staining reflective of the dead cells was clearly evident (Figure 2B), thereby confirming the cell killing observed in resazurin reduction assays (Figure 2A). Further, a fluorogenic dye DCFDA that measures the reactive oxygen species (ROS) activity within the live cells was applied; the DCFDA staining was significantly decreased at 24 h of KSS19 treatment (Figure 2B, last panel), again validating the cell elimination. Clonogenic cell survival assays to determine the effect of KSS19 on colony formation of HCT116 and HT29 cells were also performed. We found that KSS19 greatly reduced the number and size of the tumor cell colonies as represented and quantitated in Figure 2C. Together, these data show it that KSS19, as a single agent, exerts strong anticancer effects against colon cancer cells and has the ability to overcome the resistance against CA4 (perhaps against other anti-mitotics) in the HT29 type of colon cancers.

### KSS19 inhibits *in vitro* tubulin polymerization

**Figure 3:**
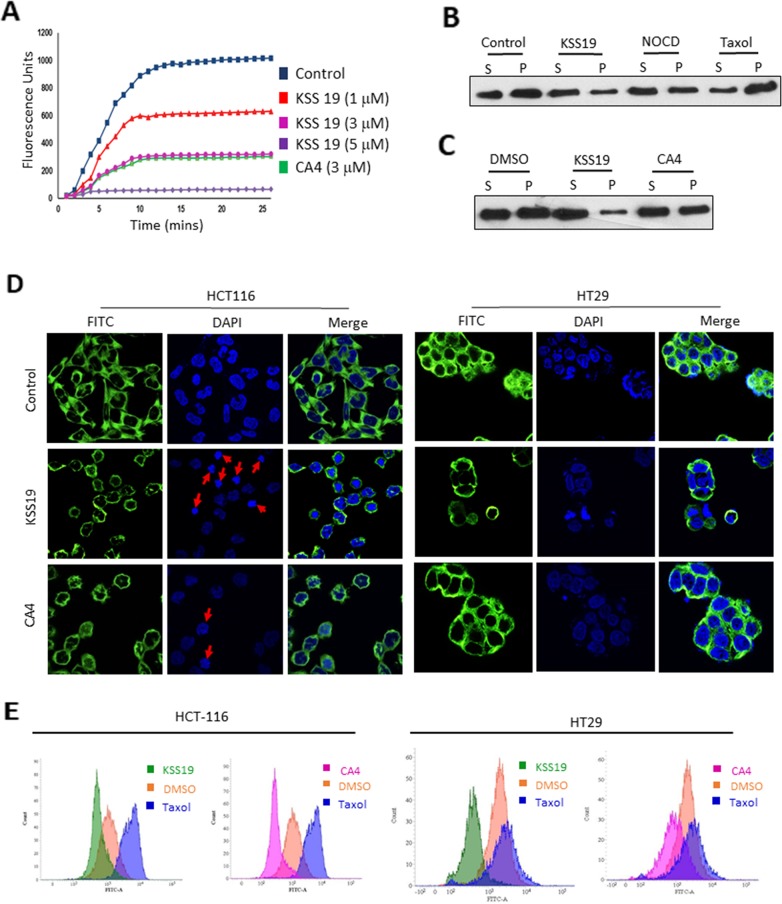
Effect of KSS19 on dynamics and structure of microtubules **(A)** Inhibition of microtubule polymerization by KSS19 *in vitro*. Time-dependent polymerization of purified tubulins in the presence of a fluorescence tag at 37°C in the presence of vehicle (DMSO), KSS19 (1 μM, 3 μM, and 5 μM), and CA-4 (3 μM) was monitored continuously by recording fluorescence at 460 nm over 30 min as described in Methods. **(B, C)** Distribution of tubulin in polymerized vs. soluble fractions analyzed by immunoblotting in KSS19 treated HT29 cells. HT29 cells were treated with 100 nM of KSS19 for 24 h. Nocodazole, paclitaxel, and CA4 were added at 2.5 μM, 1 μM, and 3 μM concentrations respectively for 24 h. The fractions containing soluble and polymerized tubulin were collected and separated by SDS-PAGE. Tubulin was detected by Western blot analysis using α-tubulin antibody. **(D)** Immunocytochemical evidence for disruption of microtubules. HT29 and HCT116 cells were independently treated with 5 μM KSS19 for 24 h. 50 nM and 3 μM CA4 was used against HCT116 and HT29 cells. The cells were then fixed and stained for tubulin. DAPI was used as a counterstain. The merged images of cells stained for tubulin and DAPI are also shown. **(E)** The whole cell flow-cytometric analysis of tubulin polymerization following 24 h of drug treatments in HT29 and HCT116 Cells. KSS19 and taxol were used at 5 μM and 1 μM respectively.

To clarify the molecular targets of KSS19, we first examined its effect on microtubules and to assess the direct interaction between tubulins and KSS19, a cell-free tubulin polymerization assay was used [31]. This polymerization assay kit used purified α and β tubulin proteins labeled with a fluorescent marker and followed its polymerization at 37°C in the presence of KSS19 or the CA4 as a positive control (Cytoskeleton, Inc.) by measuring the increase in fluorescence. Without drug treatments (DMSO; control), tubulin subunits heterodimerized and self-assembled to form microtubules in a time-dependent manner. Whereas the microtubule depolymerizing agent CA4 or KSS19 prevented tubulin polymerization as indicated by the decreased fluorescence. (Figure 3A). Results in Figure 3A also indicate that KSS19 inhibited tubulin polymerization in a concentration-dependent manner. The results suggest that KSS19 binds directly to tubulin proteins to curtail the polymerization.

### KSS19 inhibits tubulin polymerization in colon cancer cells

Because the inhibition of polymerization disturbs the assembly of microtubules, we analyzed the levels of soluble (unpolymerized) versus polymerized tubulin content [32] in HT29 cells after KSS19 and CA4 treatments. In addition, cells were treated with nocodazole (1 μM), a microtubule destabilizing agent, and paclitaxel (1 μM), a microtubule stabilizing agent, in parallel experiments. Immunoblot analysis of soluble (containing free tubulin) and insoluble (containing tubulin from microtubules) fractions revealed that the amount of tubulin protein in both soluble and polymerized fractions was approximately the same (~1:1 ratio) in untreated or DMSO treated cells (Figure 3B). In cells treated with nocodazole, as expected, there was a shift in the tubulin balance, with more amount of tubulin present in the unpolymerized fraction. Cells treated with paclitaxel, a microtubule stabilizer, displayed the reverse proportion, with more tubulin present in the polymerized fraction. KSS19 treated cells showed most of the tubulin in the unpolymerized fraction, demonstrating that KSS19 destabilized the microtubule dynamics, in a manner similar to nocodazole. Furthermore, KSS19 was more potent in altering microtubules relative to CA4 in HT29 cells with a distinct shift of the tubulin towards unpolymerized fraction as shown in Figure 3C.

Next, we analyzed the effect of KSS19 on the microtubule integrity in both HT29 and HCT116 cells after 24h treatment by immunocytochemical staining with tubulin antibodies. The untreated controls revealed a clear and organized microtubule distribution (Figure 3D). Both HT29 and HCT116 cells treated with 100 nM KSS19 showed a clear decrease in intact microtubules, with dispersed and unpolymerized structures, whereas CA4 inactive against HT29 cells, displayed a normal microtubule organization even at 1 μM. However, both KSS19 and CA4 were equally potent in destabilizing the microtubule architecture in HCT116 cells. Furthermore, we quantified the extent of tubulin polymerization in cells using flow cytometry by staining with α-tubulin antibody; taxol as a microtubule stabilizer was used the control [33]. This technique allows a rapid and quantitative analysis of polymerized tubulin biomass, enabling an easy comparison of compounds that affect tubulin polymerization, and an evaluation of the rate at which the tubulin dynamics is perturbed. Both HT29 and HCT116 cells were treated with KSS19, CA4 or taxol for 24 h, stained with α-tubulin antibody and analyzed by flow cytometry. As shown in Figure 3E, KSS19 as microtubule destabilizer decreased the fluorescence when compared to untreated cells and taxol increased the fluorescence as it promoted the tubulin polymerization. Results indicated that both KSS19 and CA4 equally potent against HCT116, but CA4, as in previous experiments was inactive in inhibiting tubulin polymerization in HT29 cells relative to KSS19.

### Molecular modeling studies

**Figure 4:**
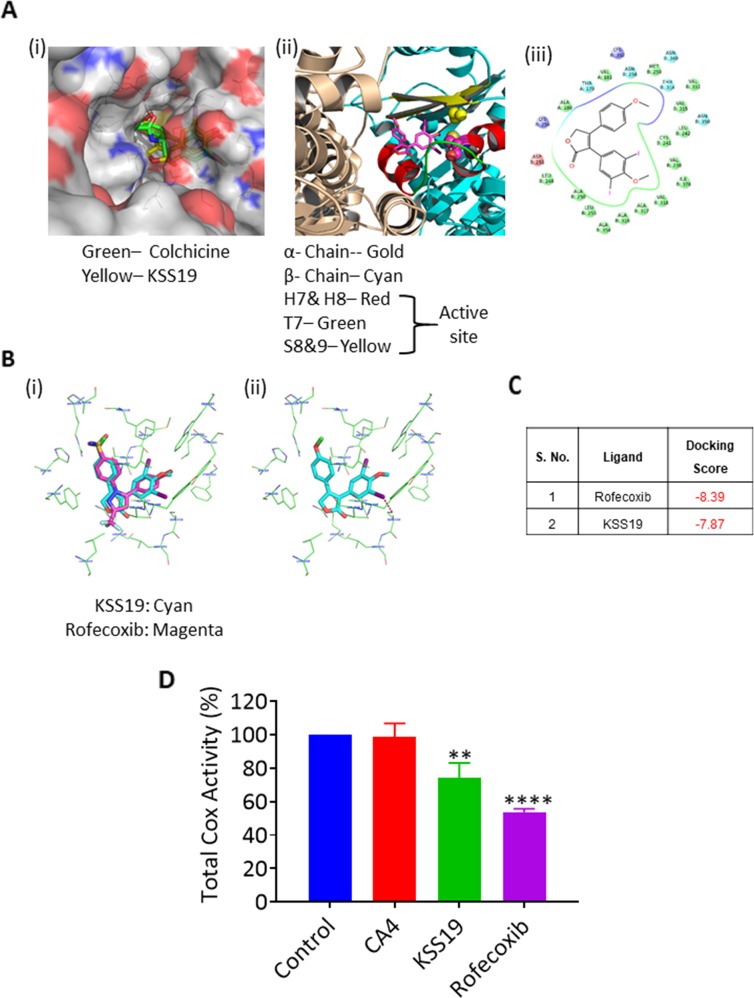
Molecular docking and affinity of KSS19 with tubulin and COX-2 proteins **(A)** Predicted binding mode of KSS19 in the tubulin colchicine binding site, which is located between the α and β subunits and mostly buried in β-subunit. The molecule KSS19 (Yellow) binds to the tubulin in a manner similar to Colchicine (green) (i). Ribbon structure showing the contribution of helices (H7 & H8), the T7 loop, and the β-strands (S8 & S9) contribution to the binding site and the interaction of KSS19 and therewith; (ii). Interaction of KSS19 with the amino acids present at the colchicine binding site (iii). **(B)** The molecule KSS19 (cyan) bound very similar to rofecoxib (magenta) (i). Predicted binding mode of KSS19 in the ligand binding domain of COX-2 (ii). **(C)** Docking scores of KSS19 and rofecoxib for the COX-2 protein. (D) Total cyclooxygenase activity was measured after treating the HT29 cells with DMSO (control), CA4 (1 μM), KSS19 (250 nM) or rofecoxib (150 nM) for 24 h as described in Methods.

To rationalize the potential binding modes of KSS19 in tubulin, docking studies were carried out by using the reported X-ray structure of tubulin co-crystallized with a colchicine derivative, N-deacetyl- N-(2-mercaptoacetyl)colchicine (DAMA-colchicine, PDB entry 1SA0). The colchicine-binding site in tubulin is mainly buried in the β-subunit while maintaining few interactions with the α-subunit; there is one such site on each tubulin heterodimer. Because of the structural similarity between the KSS19 and colchicine site ligand CA4, it was proposed to bind with tubulin at the colchicine binding site. To elucidate their mode of binding with tubulin, molecular docking studies were performed on KSS19 against the tubulin colchicine binding site. The results indicated that KSS19 binds to the tubulin protein in a manner very similar to Colchicine (green) (Figure 4A(i)). Autodock results suggest that KSS19 binds to tubulin with good affinity and a docking score of −7.30. The docking position of the 3,5-diiodo4-methoxy group of KSS19 adopt an orientation very similar to that of the trimethoxy ring of DAMA-colchicine in the co-crystallized structure and the furyl moiety was well lodged between Ala250 and Lys254. The binding of KSS19 is shown in Figure 4A(ii); where, the α and β subunits are shown in golden and cyan colors respectively. The helix H7 and H8 are shown in red, the T7 loop is shown in green and strands S8 and S9 are shown in yellow. The iodomethoxyphenyl ring goes in a cavity lined by residues Cys241, Leu248, Leu255, Val318, Val238, and Ile378. The residues Cys241 (H7) and Val318 (S8) are known to be important for binding of colchicine type ligands to tubulin. It is important to note that the oxygen atom of the methoxy group is within hydrogen bonding distance of Cys241. The other aryl ring is settled in the cavity having residues Asn258, Met259, Lys352 and Val351 (Figure 4A(iii)).

Rofecoxib, whose structure is reflected in the compound KSS19 is a potent COX-2 inhibitor. We used molecular modeling to evaluate whether KSS19 binds to COX-2 in a similar fashion to rofecoxib. The X-ray crystal structures of COX-2 in complex with rofecoxib (PDB ID: 6COX) was used to study the KSS19 interaction with the COX-2 active site. The molecule KSS19 bound in a way similar to Rofecoxib (magenta) (Figure 4B(i)) to COX-2 protein. The central furan ring sits near residues Arg120, Leu531, Ala527, Val349. The Arg120 is making a hydrogen bonding interaction with the oxygen atom of the furyl ring. The iodomethoxyphenyl ring of KSS19 goes in a cavity lined mostly by hydrophobic residues viz. Tyr348, Phe381, Leu384, Tyr385, Trp387, Met522, Leu552, and Ser530. The increased steric bulk due to the presence of iodines and methoxy group forced the ring slightly toward Ser530 as compared to the rofecoxib. The iodine may have a favorable electrostatic interaction with the hydroxyl group of Ser530. The other aryl ring is settled in the cavity lined by hydrophobic and a few polar residues viz. Tyr 355, Val523, Leu352, His90, Gln192, Arg513, Ala516, Ile517 and Phe518. The Tyr355 appears to make a π- π interaction with this ring which may help stabilize the molecule in the cavity (Figure 4B(ii)). Docking scores of both rofecoxib and KSS19 against COX-2 are presented in Figure 4C. Despite a similar binding, however, KSS19 appeared to be less potent than rofecoxib in curtailing the total Cox activity in HT29 tumor cells (Figure 4D).

### Inhibition and loss of COX-2 protein after KSS-19 treatment in colon cancer cells

**Figure 5:**
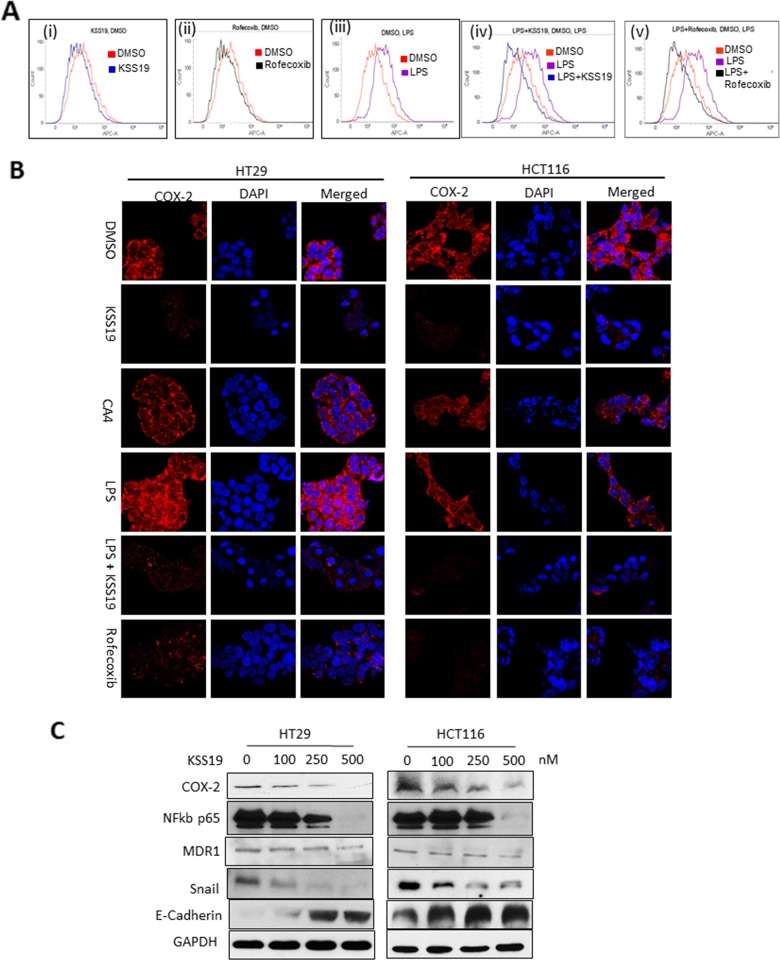
Effect of KSS19 on COX-2 in colon cancer cells **(A)** Flow cytometric analysis of HT29 cells and peak shifts observed after drug treatments. untreated (red) or KSS19 (250 nM, blue) (i); rofecoxib (150 nM, black) (ii); LPS (1 μg/mL, 24 h, Lavender) (iii); LPS + KSS19 (blue) (iv); LPS + rofecoxib (black) (v); using COX-2 specific antibody staining. **(B)** Effect of KSS19 on both basal and LPS induced COX-2 levels in HT29 and HCT116 cells analyzed by confocal immunofluorescence. Cells were incubated with DMSO (control) or KSS19 (250 nM) or CA4 (1 μM) for 12 h and COX-2 levels were visualized using COX-2 antibody immunostaining. To show the effect on LPS induced COX-2 levels, cells were pretreated with LPS (1 μg/mL) for 30 min before adding KSS19 (150 nM), and incubated for 12 h. COX-2 levels were visualized with fluorescence microscopy after immunofluorescence staining with the COX-2 antibody (red). Cells were stained with DAPI for visualization of the nuclei. Clearly evident the reduction and elimination COX-2 protein. **(C)** HT29 and HCT116 cells were treated with KSS19 for 12 h and cell lysates were subjected to immunoblot analysis with antibodies directed against COX-2, NF-kb p65, Snail, and E-Cadherin. GAPDH served as a loading control.

COX-2 has been shown to play a key role in cancer progression, by increasing proliferation of mutated cells, and favoring tumor promotion and rendering drug resistance through induction of regulatory proteins such as the NF-κb, MDR1 [34]. In view of the structural similarity of KSS19 with rofecoxib and its binding with COX-2 (Figure 2), we hypothesized that the increased cell killing of KSS19 in COX-2 overexpressing HT29 cells may result from COX-2 inhibition. To prove the inhibition of COX-2, we performed flow cytometry in permeabilized HT29 cells using COX-2 specific antibody. As shown in Figure 5A, KSS19 greatly decreased the number of COX-2 positive cells at its IC_50_ concentration similar to rofecoxib. For further verification, we stimulated the COX-2 levels using LPS (Figure 5 A(iii)) and found that KSS19 decreased even the elevated COX-2 levels with a distinct shift in fluorescence peak indicating its potential use for inhibiting the overexpressed COX-2 in CRC (Figure 5A (iv and v)). Next, both HT29 and HCT116 cells were treated with KSS19 and immunofluorescence experiments were performed using the COX-2 specific antibody. Rofecoxib was used a positive control and representative photomicrographs from these experiments are shown in Figure 5B. KSS19 induced marked decrease in the immunofluorescence of the COX-2 as potently as rofecoxib. In contrast, KSS19 parent molecule CA4, as expected, had no effect on COX-2, both in HCT116 and HT29 cells indicating the hybrid drug nature of KSS19. The inactivated COX-2 appears to undergo degradation, and this was confirmed at the protein level by Western blotting. Treatment of both HT29 and HCT116 cells with KSS19 caused a dose-dependent decrease in the COX-2 protein levels (Figure 5C). Since COX-2 regulates the expression of MDR1 levels through c-Jun at Ser63/73 [19], KSS19 also reduced the levels MDR1, which is overexpressed in colon cancer cells in a dose-dependent manner. Further, the KSS19 increased the levels of E-cadherin along with a drop in the NF-κB and Snail protein levels (Figure 5C). Since COX-2 regulates the E-cadherin expression through the NF-kB: Snail signaling pathway, and in turn augment cancer invasion and metastasis [35], our observations are significant. E-cadherin is essential for the cell to cell attachment. The Snail transcription factor is required for epithelial-mesenchymal transition (EMT) [36]. Thus, our data that KSS19 reduces the NF-κb and the EMT marker snail along with an increase in E-cadherin levels reflects the utility of the hybrid drug in attenuating CRC metastasis.

### KSS19 inhibits cell migration

**Figure 6:**
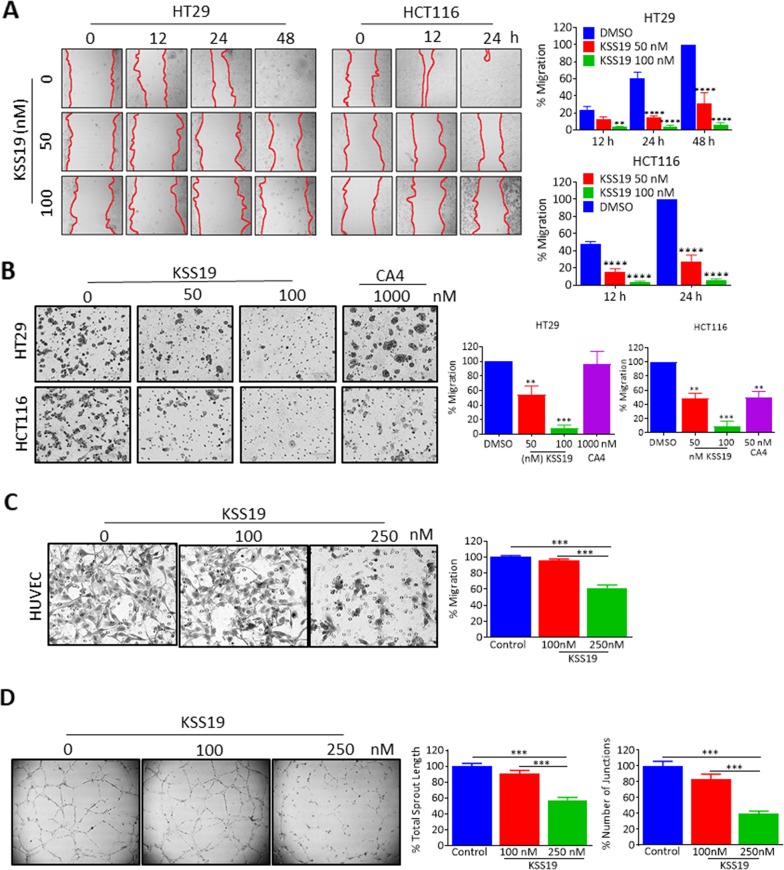
KSS19 inhibits cancer cell migration and tube formation in HUVE cells **(A)** HT29 and HCT116 cells were grown to confluence in six-well plates and a scratch was made at experimental time zero. Then the cells were exposed to various concentrations of KSS19. The wells were imaged at different time points and the migration quantified. Results shown are average values of 6 representative fields in each of two different experiments performed in duplicates, and error bars represent SEM. **(B)** KSS19 inhibits HT29 and HCT116 cell migration. HT29 and HCT116 cells were subject to transwell migration assay 12 h after treatment with KSS19 (0, 50 and 100 nM) and CA4 (1 μM) as specified. The crystal violet dye staining images of lower chambers with migrated cells are shown. The right panel shows the data as mean + SE of a number of cells that migrated in 3 different experiments ^**^P<0.01 and ^***^P<0.001 as compared with the controls. **(C)** Efficient inhibition of endothelial cells migration by KSS19. Serum-starved HUVECs in ECM containing 0.5% FBS were pretreated with KSS19 (0, 100 and 250 nM) for 6 h. These cells were then seeded in the upper chamber of Transwells and allowed to migrate to the lower chamber with 500 μl ECM containing 0.5% FBS and 50 ng/ml VEGF. After 5 h incubation, non-migrated cells were removed with cotton swabs, and migrated cells were fixed with cold 4% paraformaldehyde and stained with 1% crystal violet. Images were taken using an inverted microscope (Olympus; magnification, 100), and migrated cells in random 4 fields were quantified by manual counting. Three independent experiments in triplicate were carried out. The right panel shows the data as mean + SE of a number of cells that migrated in 3 different experiments ^**^P<0.01 and ^***^P<0.001 as compared with controls. **(D)** KSS19 remarkably inhibited the formation of endothelial tubular structure. HUVEC cells were seeded into Matrigel-coated 98 well plate and treated with 100 and 250 nM KSS19 after 24 h images were taken from a microscope. The right panel shows the data as mean + SE of total sprout length and number of junctions in 3 different experiments (^***^P<0.001) as compared with controls.

Metastasis involves multiple steps including cancer cell motility, intravasation, transit and survival in circulation, extravasation and growth at the new site [37]. Encouraged by the results obtained hitherto, we examined the effects of KSS19 on the ability of both HT29 and HCT116 cells migration and invasion *in vitro*. In the scratch wound healing assay, cells along the wound-edge migrate into the nude space after scratching and the scratch-wound would heal continuously. Untreated cells had no evident effect on the migration and wound was healed in 48h in case of HT29 and 24h in HCT116. KSS19 at low concentrations of 100 nM prominently inhibited both HT29 and HCT116 cancer cell migration and curtailed the wound healing rate in a dose-dependent manner (Figure 6A). Statistical analysis demonstrated that KSS19 at lower concentrations significantly decreased the wound repair in both HT29 and HCT116 cells (Figure 6A). In addition, to determine the effects of the KSS19 on cell migration, we performed transwell migration assays in HT29 and HCT116 cells. As shown in Figure 6B, KSS19 markedly inhibited the migration activity with greater than 50% and 80% inhibition against 50 nM and 100 nM respectively (Figure 6B). Together, these data demonstrate that KSS19 has the ability to suppress the migration/invasion of CRC at low concentrations.

### Evidence for the anti-angiogenic activity of KSS19

Angiogenesis is a complex process that includes the proliferation, migration and tube formation of vascular endothelial cells [38]. Agents that target the microtubule cytoskeleton, which is essential for the cell movement are reported to interfere with angiogenesis [39]. In addition, COX-2 inhibition increases the expression of E-cadherin which promotes cell adhesion [40]. Since KSS19 contains the pharmacophore of CA4, which is well known to be anti-angiogenic [41], we examined the vascular targeting and anti-angiogenic properties of the hybrid drug both at the cell culture and xenograft levels. For this, first, we tested the inhibitory effect of KSS19 on HUVEC endothelial cell proliferation by resazurin reduction assay and found growth inhibition at 365 nM. Therefore, concentrations below 365 nM of KSS19 were used to assess the anti-angiogenic effects in HUVECs according to a previous procedure [38]. HUVEC migration was investigated in a transwell system, in which cells were allowed to invade through Matrigel in the direction of a chemoattractant (recombinant human vascular endothelial growth factor) for 24 h. In agreement with the results obtained from the above migration assay of colon tumor cells (Figure 6A and 6B), KSS19 proved to be an effective inhibitor of HUVEC migration. When the migration of untreated cells was set at 100%, only 60% and 46.0 ± 8.2% (mean ± SE) cell migration was observed after 100 nM and 250 nM of KSS19 treatments respectively (Figure 6C).

Subsequently, we investigated the activity of KSS19 on preformed HUVEC endothelial capillaries (Figure 6D). Endothelial cells were plated on matrigel and allowed to form capillary tubes for 22 h, followed by exposure to the KSS19 at 100 and 250 nM concentrations. Microscopic quantification of total capillary length and number of joints at the 0h, 6h and 24h were performed. KSS19 triggered a concentration-dependent decrease in the total tube length and number of joints. At 6-hour time point, there was a 22% decrease in the tube length when compared to DMSO control and a 65% decrease was observed at 24h with 250 nM of KSS19.

### Effect of KSS19 on molecular events of cell cycle progression

**Figure 7:**
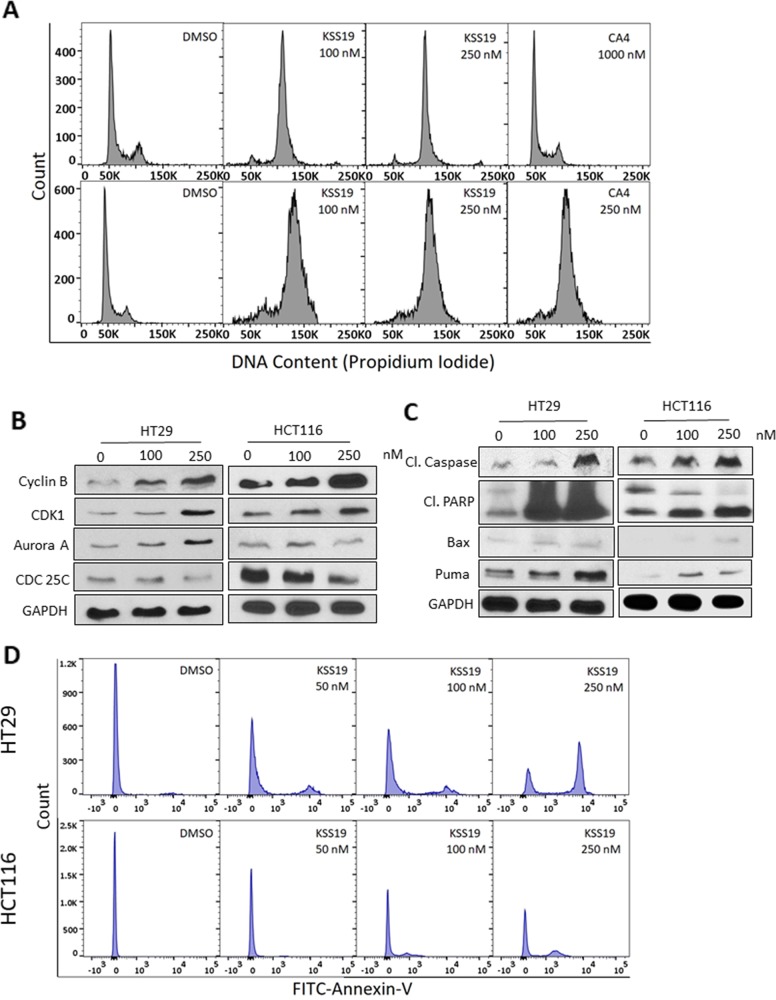
KSS19 induces G2/M cell cycle arrest and apoptosis **(A)** Flow cytometric analysis of the KSS19 effect on cell cycle progression. HT29 and HCT116 cells were treated with KSS19 or CA4 or DMSO as vehicle control for 24 h. Cells were stained with propidium iodide and analyzed. A strong G2/M phase block is evident. The percentage of cells in each phase of cell cycle was quantified. **(B)** Effect of KSS9 on the expression of cyclin B1, aurora kinase A, CDK1 and CDC25C levels by immunoblotting in both HT29 and HCT116 cells. Cells were treated with 100 and 250 nM of KSS19 for 24 h and compared with control. **(C)** Western blot analysis of the intrinsic apoptotic molecules in KSS19 treated HT29 and HCT116 cells show enhanced expression. **(D)** Apoptosis analysis by flow cytometry using Annexin V-FITC stain assay. HT29 and HCT116 cells were incubated with various concentrations of KSS19 for 24 h. To compare apoptosis, FITC-conjugated annexin binding to phosphatidylserine, exposed to the outer surface, on treatment with KSS19 was measured by FACS.

Microtubule-disrupting agents induce a mitotic arrest because of their essential role in spindle assembly and chromosomal segregation [42]. Consequently, the impact of KSS19 on cell cycle progression was determined in HT29 and HCT116 cells by flow cytometry (Figure 7). Untreated cells showed an asynchronous pattern of proliferating cells largely distributed in the G1 (55.2%), S (32.4%), and G2/M (12.4%) phases. KSS19 at 100 nM elicited a clear G2/M arrest pattern in both HT29 and HCT116 cells with a concomitant decrease of cells in other cell cycle phases (Figure 7A). This result is similar to other microtubule-interacting agents. However, CA4 was not able to induce a G2/M arrest in HT29 cells even at higher concentration, whereas it was equally potent to KSS19 in inducing this cell cycle blockade in HCT116 cells. These data are consistent with a high-level resistance of HT29 cells to CA4 and the strong efficacy of KSS19 against this cell type.

Next, we determined the association between KSS19 induced G2/M arrest and alterations in the expression of proteins that regulate cell division. The major regulator of the G2 to M transition is cyclin B/CDK1 [43]. This master CDK is in an inactive state by phosphorylation of CDK1 at two negative regulatory sites (Thr14 and Tyr15), which are dephosphorylated by cdc25c phosphatases. CRC treatments with KSS19 for 24 h induced a concentration-dependent accumulation of cyclin B1 and CDK1 and attenuation of Cdc25C levels in both HT29 and HCT116 (Figure 7B). Aurora Kinases, which are routinely expressed during the G2/M phase of cell cycle function to regulate the microtubule-chromosome interactions, spindle stability and cytokinesis [44]. In accordance, the Aurora kinase A levels increased in cells arrested at G2/M after KSS19 treatment.

### KSS19 induces apoptosis

**Figure 8:**
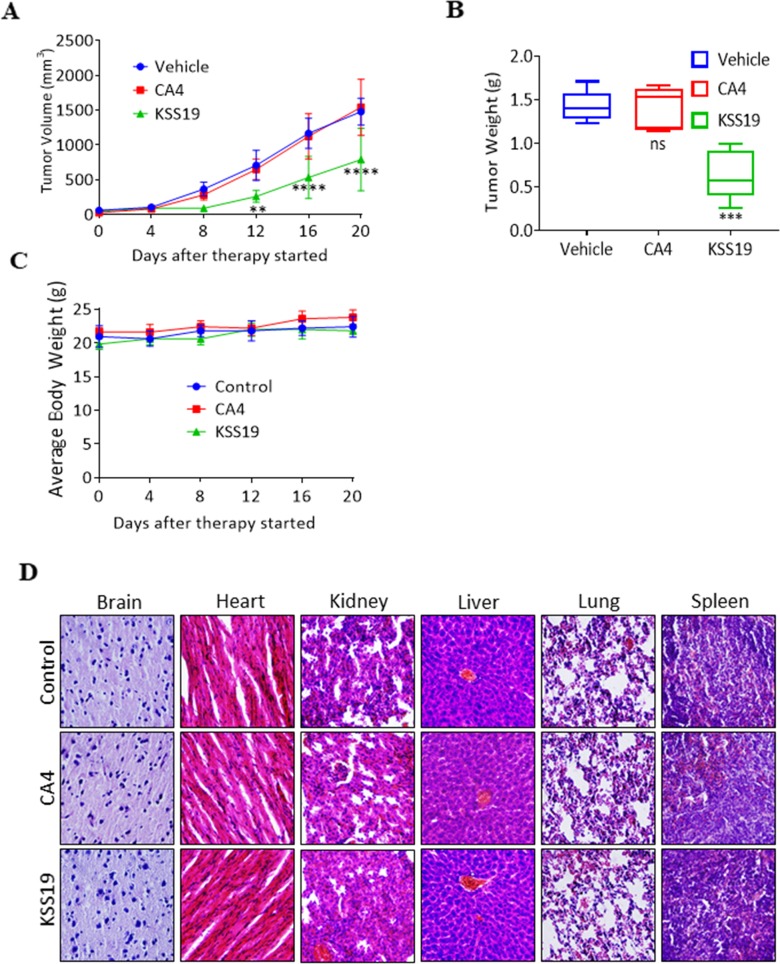
KSS19 suppresses colon tumor xenograft growth rate in nude mice **(A)** Nude mice bearing HT29 xenografted tumors were administered with KSS19 and CA4 by i.p. injections at 25 mg/kg per day, 5 days per week for 20 days. The tumor volumes were calculated and plotted. **(B)** At the end of the experiments, tumors were excised and weighed. **(C)** Animals were also monitored for changes in body weight as a surrogate marker of toxicity. **(D)** H&E stained tissue sections of major organs including liver, spleen, lungs, kidneys, heart, and brain of the nude mice treated with KSS19, CA4, and vehicle alone. No major histological changes were observed after drug treatments.

The mode of cell death induced by KSS19 was quantitated by FITC labeled Annexin-V method which stains the phosphatidylserine (PS) residues in early apoptotic stages [45]. Both HT29 and HCT116 cells were labeled with Annexin-V-FITC after KSS19 treatment and subjected to flow cytometry. From the Figure 8A, it is clear that KSS19 triggered an accumulation of apoptotic cells in a concentration-dependent fashion after 24 h treatment. Furthermore, consistent with the observed apoptosis, the players involved, namely, the cleaved caspase-3 and cleaved PARP (poly ADP-ribose polymerase) were expressed at higher levels in KSS19 treated cells, again, in a concentration-dependent manner. In addition, the apoptotic proteins BAX and PUMA were also upregulated following KSS19 exposure.

### *In vivo* antitumor activity of KSS19 in a mouse subcutaneous xenograft model

To test whether KSS19 affects the growth of solid tumors *in vivo*, we evaluated its anticancer activity in HT29 xenografts developed in nude mice. Exponentially growing HT29 cells were injected s.c. into mice. When the tumors reached 60 mm^3^, the mice were given KSS19 or CA4, through i.p. injections and monitored the tumor growth. KSS19 at 25 mg/kg/day inhibited tumor growth by 55% (P < 0.01), whereas the same dose of CA4 did not show any significant effect when compared to vehicle alone controls (Figure 8A). KSS19 also effectively reduced the overall tumor weight when compared to the vehicle or CA4 treatment (Figure 8B). Of note, there were no remarkable changes in the average body weights in either model, suggesting that the treatment did not lead to overt toxicity (Figure 8C). Further, there were no significant differences in the histological findings among the treatment and control groups in any of the tissues examined including the liver, kidney, heart, spleen, and brain (Figure 8D); these data indicate that KSS19 is unlikely to exert any adverse effects in host organs and tissues at therapeutically relevant doses.

**Figure 9:**
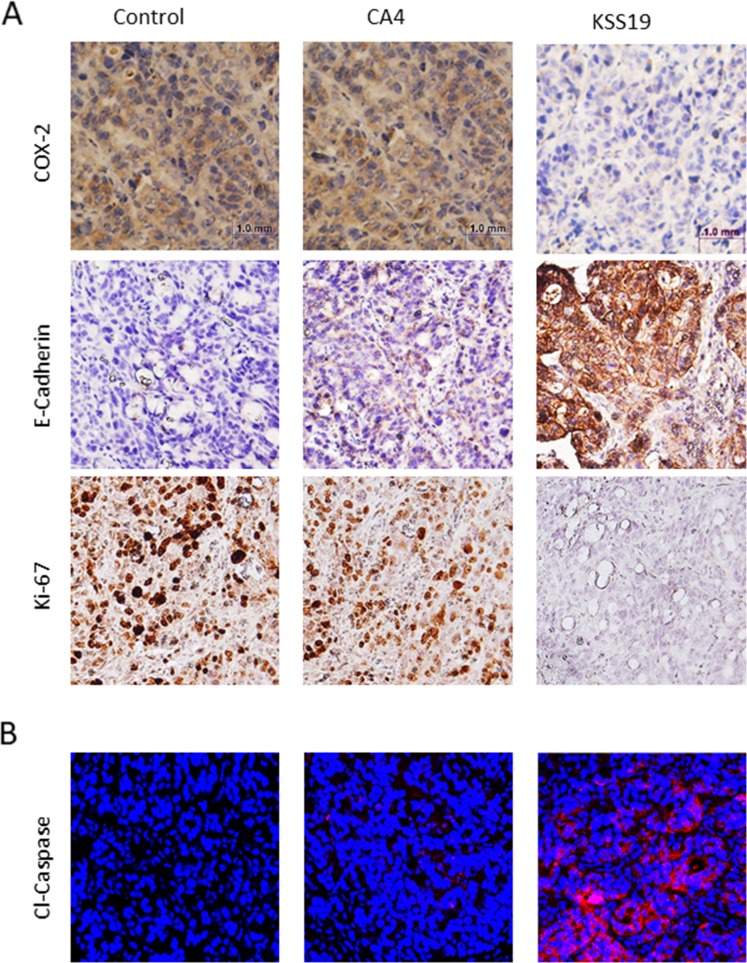
Effects of KSS19 and CA4 on COX-2, E-cadherin, cell proliferation and apoptosis in HT29 xenografted tumor tissues **(A)** Representative images of COX-2, E-cadherin and Ki67 immunostaining in formalin-fixed, paraffin-embedded HT29 tumor tissues from control and KSS19 and CA4 administered nude mice. **(B)** Apoptosis induced by KSS19 as indicated by the increased expression of cleaved caspase-3 expression on the cryosections of tumors after treatment with KSS19 relative to the vehicle alone or CA4 treatment.

**Figure 10:**
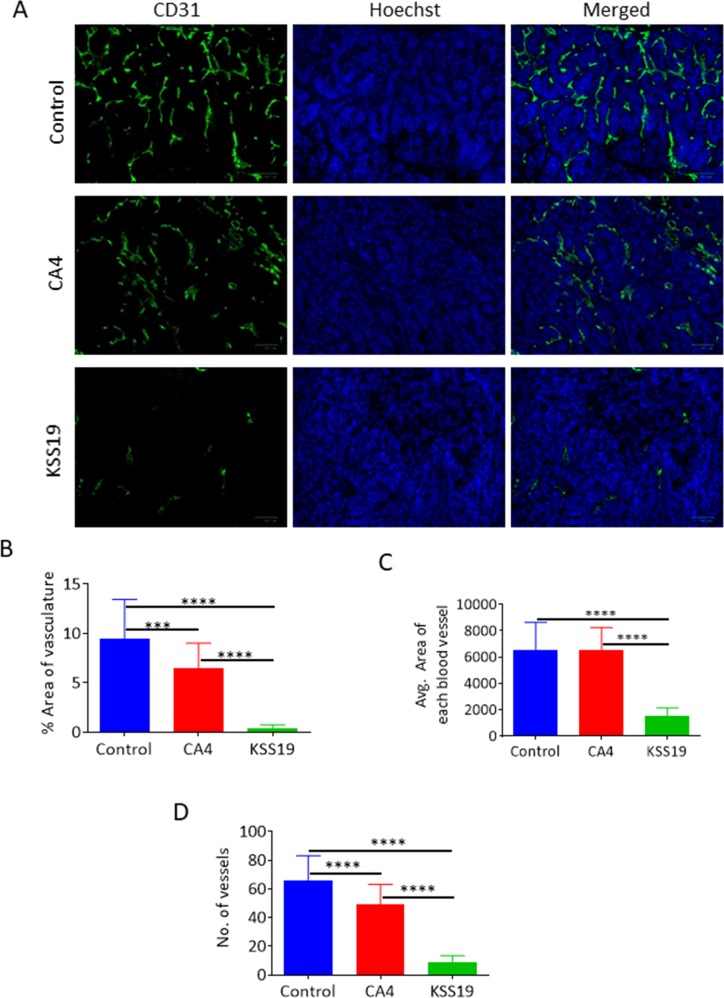
Effects of KSS72 and CA4 on HT29 tumor angiogenesis **(A)** Representative CD31 staining of HT29 xenograft tumor sections, showing a reduction in the number of functional vessels in tumors treated with KSS19. CA4 was less efficient than KSS19 in curtailing the angiogenesis in this model. **(B-D)** Quantification of the overall vessel density (total number of vessels), vascular area and the average area of each blood vessel. Data represented as mean ± SE of 3 different tumors (^***^P<0.001) and compared with vehicle-treated tumors.

To further demonstrate the effect of KSS19 on COX-2, E-cadherin, Ki-67, and angiogenesis *in vivo*, we evaluated the blood vessel formation, expression levels of the proteins mentioned above in the excised HT29 xenografted tumor sections using immunohistochemical (IHC) procedure. As shown in Figure 9, the intratumoral COX-2 protein levels were significantly reduced after KSS19 administration, whereas no effect was observed with CA4. In line with aforementioned *in vitro* results, COX-2 inhibition elevated the cell surface expression of E-cadherin compared to control. In addition, we found a decreased cell proliferation reflected by attenuated expression of the Ki-67 marker in tumor tissues. Moreover, we observed significantly higher levels of cleaved caspase-3, an apoptotic marker, in the KSS19-treated group. Next, we measured microvessel density at the periphery of the tumors using CD31 staining to demonstrate the effect of KSS19 on the angiogenesis (Figure 10). CD31 is a well-characterized endothelial cell marker used to evaluate the degree of tumor angiogenesis in histological tissue sections [46]. KSS19 effectively inhibited the angiogenesis by reducing the number of blood vessels, average area of blood vessels and percentage of the vasculature. Nevertheless, there was no significant change in the formation of blood vessels in CA4 administered HT29 tumors relative to the untreated controls. Taken, together, these results demonstrate a reduced angiogenesis in HT29 xenografts following KSS19 administration.

## MATERIALS AND METHODS

### Cell lines and cell culture

Human colorectal cancer cell lines HT29, HCT116, SW620 and human umbilical vein cells (HUVEC) were purchased from the American Type Culture Collection (Rockville, MD, USA). All cancer cell lines were grown in Dulbecco's modified Eagle's (DMEM) medium containing 10% Fetal Bovine Serum and 1% penicillin/streptomycin in a humidified atmosphere of 5% CO_2_ at 37^°C^. HUVECs were grown in M199 medium, supplemented with 15% fetal bovine serum, 150 mg/ml endothelial cell growth supplement, 5 U/ml heparin sodium and 1X antibiotic/antimycotic solution (Gibco).

### Chemicals, antibodies, and other reagents

CA4 is purchased from Santacruz. All other chemical and reagents were obtained from Fisher Scientific Company. Antibodies to cleaved PARP, cleaved caspase, cyclin B1, aurora kinase A, CDK1 and CDC25C, COX-2, NF-κβ, E-Cadherin and Snail proteins were purchased from Cell Signal Technology.

### Synthesis of KSS19

To a mixture of 2-(3,5-diiodo-4-methoxyphenyl)acetic acid **1** (417 mg, 1 mmol), 8 mL of acetonitrile and (253 mg, 1.2 mmole) of 2-bromo-1-(4-methoxyphenyl)ethan-1-one **2**, triethyl amine (0.2 mL) was added drop wise and under argon. The resulting mixture was stirred for 1 hour at room temperature and then cooled to 0°C followed by adding 0.2 mL of diazabicyclo[5.4.0]-undec-7-ene (DBU) and the mixture was stirred for another 2 hours at this temperature. Then 10 mL of 1 N hydrochloric acid was added and the mixture was extracted with ethyl acetate. The organic extract was dried over anhydrous Na_2_SO_4_ and concentrated under reduced pressure. The residue was purified by column chromatography using ethyl acetate/hexane to give KSS19 (350 mg, 63%) as a pale-yellow color solid. 1H NMR (300 MHz, CDCl3): δ 3.81 (s, 3H), 3.84 (s, 3H), 4.97 (s, 2H), 6.89 (d, 2H, *J* = 7.3 Hz), 7.36 (d, 2H, *J* = 7.3 Hz), 7.81 (s, 2H). 13C NMR (75 MHz, CDCl3): 55.6, 60.5, 74.8, 87.2, 114.2, 129.2, 129.8, 131.1, 131.7, 143.7, 159.2, 159.8, 168.9. ESI MS (*m/z*): 549 (M + H).

### Cytotoxicity assays

KSS19 was evaluated for *in vitro* cytotoxicity in human colorectal cancer cell lines following a protocol of 72 h continuous drug exposure using resazurin reduction assay. The cell lines were grown in 96-well microtiter plates at a density of 5000 cells per well. The plates were incubated for 24 prior to addition of drug. Nine concentrations of KSS19 were evaluated in quadruplicate sets. Plates were incubated for a further 72 h and 20 μl resazurin 0.01% (w/v) was added to each well. After 2 h, the fluorescence was measured using a Tecan Reader (Infinite m200 Pro) at a 544 nm excitation and 590 nm emission.

### Cell viability assays using PI and DCFDA

We evaluated KSS19 for cytotoxicity and viability using PI and DCFDA staining in HT29 and HCT116 cells. Approx. 1×10^6^ cells were plated in each well in duplicate 6 well plates and were allowed to grow. Then KSS19 and CA4 were added at different concentrations to the wells and cell growth was continued for 24 hours. Then to one set of the plates, DCFDA (20μM dissolved in DMSO) was added followed by incubation for 30 min, media removal, and visualization under a fluorescent microscope. For the second set of plates, media was removed and then PI (1mg/ml in PBS) was added, incubated for 15 min, followed by media removal and fluorescence microscopy.

### Clonogenic survival assays

Around 500 HT29 or HCT116 cells were plated in 6 well plates and were allowed to attach for 24 hours. Cells were then treated with either DMSO vehicle or different concentrations of KSS19 or CA4 for 24 h. Next, the media was removed and cells were washed once with 1X PBS. Then fresh DMEM medium was added allowed incubated for 7 days. The cells were fixed in ice-cold methanol for 15 min and washed with PBS. Crystal violet (0.5%w/v) dissolved in PBS was added and cells were stained for 1 h. The stain was removed by washing with PBS and cells were photographed under a microscope. The images were quantitated using the Image J software. Results obtained from three separate experiments were statistically validated.

### *In vitro* tubulin polymerization assay

The *in vitro* time-dependent polymerization of tubulin to microtubules was followed using a fluorescence-based tubulin assay kit (BK011, Cytoskeleton, Inc.) The reaction mixture in a final volume of 10 μl in PEM buffer (80mM PIPES, 0.5mM ethylene glycol tetraacetic acid (EGTA), 2 mM MgCl_2_, pH 6.9) contained 2 mg/ml bovine brain tubulin, 10 μM fluorescent reporter, and 1 mM GTP with or without the drugs. Tubulin polymerization was followed at 37^°C^ by monitoring the fluorescence enhancement due to the incorporation of fluorescence reporter into microtubules as polymerization occurred. Fluorescence emission at 420 nm and excitation wavelength at 360 nm was measured for 30 min at 1 min intervals in a plate reader. Combretastatin A4 was used as positive control under similar experimental conditions.

### Immunoblot analysis of soluble versus polymerized tubulin in colon cancer cells

Tumor cell monolayers grown in small plates were treated with KSS19for 24 h. The cells were washed with PBS and were permeabilized with 200 μl of a pre-warmed buffer [80 mm PIPES-KOH (pH 6.8), 1 mm MgCl_2_, 1mm EGTA, 0.2% Triton X-100, 10% glycerol and 1 x Protease inhibitor] and incubated for 5 min at 30^°C^. The supernatants which represented the soluble fraction were carefully removed, mixed with 4X Laemmli gel sample buffer and boiled for 3 min. To collect the insoluble tubulin fraction, 250 μl of 1X Laemmli sample buffer was added to the original wells (from which the soluble fractions were retrieved). These samples containing the insoluble fraction were collected and boiled for 3 min. The protein fractions were western blotted and probed with mouse anti-human α-tubulin antibodies.

### Immunofluorescence

Cells seeded on glass coverslips were incubated for times specified in the presence or absence of test compounds. Cells were then fixed in 4% formaldehyde diluted in PBS for 15 min and washed twice. After incubation with 5% goat serum and 0.3% Triton X-100 in PBS for 1 h for blocking, the cells were incubated with the primary antibodies in a buffer (1% BSA and 0.3% Triton X-100 in 1x PBS) overnight at 4°C. Next, they were washed with PBS and then incubated with FITC-conjugated secondary antibody for 1 h. After washing, the slides were mounted with DAPI-containing mounting medium. The images quantitated and photographed using a multiphoton fluorescence microscope.

### FACS analysis to distinguish soluble and polymerized tubulin fractions

About 1×10^6^ HT29 and HCT116 cells were treated with the test compounds for 24 h. The trypsinized cells were washed and pelleted in 2 ml microfuge tubes. They were fixed by adding 1 ml of 0.5% glutaraldehyde in Microtubule Stabilizing Buffer (MTSB: 80 mM Pipes [pH 6.8], 1 mM MgCl2, 5 mM EDTA, and 0.5% Triton X-100). Following a 10 min incubation at room temperature, glutaraldehyde was quenched by the addition of 0.7 ml of 1 mg/ml NaBH_4_ in PBS. After washing and blocking in antibody dilution buffer (PBS [pH 7.4], 0.2% TX-100, 2% BSA, and 0.1% NaN_3_) for 3 h, the cells were incubated with anti α- Tubulin antibody (1 μg/ml) for 3h, followed by FITC conjugated secondary antibody for 1h. The washed cells were analyzed by flow cytometry.

### Cell cycle analysis

The effect of KSS19 treatment on cell cycle progression was determined by flow cytometry following staining with propidium iodide (PI) as described previously [31]. The stained cells were analyzed by flow cytometry on a BD-FACS CantoTM II instrument.

### Cell mobility (wound heal) assays

HT29 or HCT116cells were plated in 6-well plates and were allowed to grow to 80% confluency. Using a 10 μL sterile pipette tip, two scratches were made perpendicular to each other in all wells. The scratches with cells next to them were imaged at 10X magnification at random positions and these images were considered as 0-h points. Again, images were taken at 12, 24 hours for HCT116 and 12, 24 and 48 hours for HT29 cells. Results were obtained from three independent experiments.

### Cancer cell migration and invasion assays

For the migration assays, 5 × 10^4^ cells in serum-free media were placed into the upper chamber of an insert (8-μm pore size; BD Bioscience). For the invasion assays, 2 × 10^4^ cells in serum-free media were placed into the upper chamber of an insert coated with Matrigel (BD Bioscience). Media containing 10% FBS was added to the lower chambers. Next, the test compounds at various concentrations were added and incubated for 24 h. The cells remaining on the upper membrane were removed with cotton wool, whereas the cells that had migrated or invaded through the membrane were stained with 20% methanol and 0.1% crystal violet and imaged under the microscope. The images were analyzed using ImageJ software. Experiments were repeated three times.

### HUVE cell migration assays

Migration assays were performed in 48-well micro chemotaxis chambers (Neuroprobe) through Collagen I-coated polycarbonate membranes with 8μm pores (Neuroprobe). The bottom chambers were filled with 30 μl of complete media per well with/without the tested compounds. HUVECs were harvested and resuspended at 10^6^ cells/ ml in complete media. The upper chambers were loaded with 50 μl cell suspension and incubated for 6 h at 37°C. Next, the filters were fixed with methanol and stained with hematoxylin solution. The cells that had migrated through the filter were manually quantified under an inverted microscope at 20X magnification.

### Matrigel tube formation assays

The previous procedure [47] was slightly modified. Cultrex® Basement Membrane Extract (Trevigen) was used to coat the wells of 96-well tissue culture plates (40 μl/well) and was left to polymerize for 30 min at 37°C. After this, 10^4^ HUVE Cells, suspended in 0.1 ml of complete HUVEC media, was added to each well. The test agents were added to the media on top and incubated for 6 h at 37°C. The tube networks were photographed on an inverted microscope equipped with a color digital camera. The length of the tube network, as well as the number of junctions, were determined with ImageJ analysis software (http://rsb.info.nih.gov/ij/index.html) with the Angiogenesis Analyzer plug-in.

### Measurement of tumor vessel density and size in xenografted tumor sections

Tumors were dissected from the flanks of the nude mice and were paraffin-embedded for H&E analysis or preserved in OCT medium for cryosections. At a minimum, 4 tumors from each group were embedded and serially sectioned for vessel density analysis. Immunofluorescent labeling of CD31/PECAM was done as follows: Samples were fixed with 4% PFA/PBS for 20 min, washed twice with PBS, permeabilized with Triton X-100, 0.15% + 200 mM Glycine/PBS for 15 min, washed twice with PBS, blocked with 3% BSA/PBS for 1-2 h and stained with anti CD31/PECAM (BD Biosciences #550274, 1:10) at 4°C overnight. After two 10 min washes with PBS, the slides were incubated with anti-rat Alexa 488 secondary Ab (1:500) for 2 h, followed by PBS washes. Finally, slides were counterstained with Hoechst (1:2000), washed with PBS followed by distilled water and mounted. Images were taken using a Zoe fluorescent microscope or Nikon A1 multiphoton confocal microscope with a 4x objective. Ten images were taken randomly from within the tumor body and average vessel area was measured per high power imaging field. Both the number of vessels and average vessel area were measured using ImageJ. The data were reported as a mean ± SD.

### Annexin V-FITC detection of apoptosis

Tumor cells were harvested, washed with PBS and resuspended in 1 ml of binding buffer (5x: 10 mM Hepes pH 7.4, 150 mM NaCl, 2.5 mM CaCl_2_, 1 mM MgCl_2_, 4% BSA). The cell suspensions (500 μl) were then incubated with 5 μl of Annexin V- FITC (BD Bioscience) for 30 min at room temperature. The population of Annexin V-positive cells was evaluated by flow cytometry [48].

### Molecular modeling

#### Glide docking using extra precision (XP) mode

The X-ray crystal structures of COX-2 in complex with Celecoxib (PDB ID: 6COX) and DAMA-Colchicine with Tubulin (PDB ID: 1SA0) were used for the docking of ligands in COX-2 and Tubulin respectively. Prior to docking both of the proteins were prepared in Schrodinger molecular modeling software by assigning correct bond orders, the addition of hydrogen and other missing atoms and assignment of charges using OPLS-AA force field. The added hydrogens were optimized which was followed by a restrained minimization to a gradient of 0.3 Kcal/MolÅ2 to remove any bad contacts without disturbing the active site geometry. Separate docking grids were generated using co-crystallized ligand (Celecoxib and Colchicine) as grid center with default settings for COX-2 and Tubulin respectively.

The ligands were sketched in Schrodinger molecular modeling software and were minimized to a gradient of 0.001KCal/MolÅ2. Docking was performed with Glide software using extra precision (XP) mode. The XP descriptor information was recorded and Epik state penalties were added to the docking score. The ligands were sampled as flexible. The docked poses were ranked by GlideScore.

### COX activity assay

The COX activity kit (760151, Cayman Chemicals), was used to measure the peroxidase activity of COX enzymes in cell extracts. The assay measures the formation of PGH2 by coupling the reduction of the initial peroxide product, PGG2 to the oxidation of an optically active co-substrate, N,N,N′,N′-tetramethyl-p-phenylenediamine (TMPD). Tumor cells were harvested after treating with DMSO (control), CA4 (1 μM), KSS19 (250 nM) and rofecoxib (150 nM) for 24 h. The cell pellets were homogenized in a cold buffer (100 μM Tris-HCl, pH 7.8, containing 1 mM EDTA) and the cell lysates collected by centrifugation. To these extracts, 20 μl of 2.2 mM arachidonic acid and TMPD (10 μM) were added and the total activity of Cox enzymes was assayed by monitoring the appearance of oxidized TMPD at 590 nm.

### Xenograft studies

The animal study protocol was approved by the Institutional Animal Use and Care Committee (IACUC). Female athymic pathogen-free nude mice (nu/nu, 4-6 weeks) were purchased from Charles River Laboratories (Wilmington, MA, USA). To establish HT29 xenografts, a total of 5 × 10^6^ cells (in 0.1 ml) were subcutaneously injected into the left inguinal area of the mice. All animals were monitored for activity, physical condition, body weight, and tumor growth. When the tumor volume reached ~100 mm^3^, the mice bearing HT29 xenografts were randomly divided into multiple treatment and control groups (6-8 mice/group). Test compounds were dissolved in a vehicle of composition, PEG-400: EtOH: saline (57.1:14.3:28.6, v/v/v). CA4 (100 mg/kg/d) and KSS19 (25 mg/kg/d) were administered by i.p. injections five times a week for 3 weeks. The control group received the vehicle only and tumor volume was measured using the calipers. At the end of the experiments, the xenograft tumors, hearts, lungs, livers, kidneys, spleens, and brains were removed, weighed, and snap frozen for western blotting, immunohistochemistry and immunofluorescence studies.

### Immunohistochemistry

The tissues were fixed and embedded in paraffin, cut into 5 μm sections, and then affixed to glass slides. For immunohistochemical studies, the tumor sections were blocked and incubated with a biotinylated anti-human COX-2, E-Cadherin and Ki-67 antibodies (diluted 1:50 in 5% BSA in PBS) for 1–2 h at room temperature. Sections were then incubated with pre-diluted streptavidin-peroxidase HRP conjugates and stained with DAB chromogen according to the manufacturer's instructions (DACO Animal research kit). Finally, sections were lightly counterstained with hematoxylin.

### Blood vessel assessment in tumor sections

Microvessel density (MVD) in tumor tissues was evaluated by immunofluorescence staining of CD31. Frozen 10 μm sections were thawed, fixed in acetone and blocked in 5% goat serum supplemented with 2.5% BSA. Sections were incubated overnight at 4°C in the presence of a rat anti-human CD31 antibody (dilution 1:50) (BD Biosciences). A FITC labeled goat anti-rat antibody was used as the secondary antibody (dilution 1:200) for 1 h. After washing with PBS, the slides were mounted with DAPI-containing mounting medium and photographed and images quantitated using a multiphoton fluorescence microscope. Average MVD was calculated by counting the average number of CD31 labeled vessels from ten randomized fields under the fluorescence microscope. All quantitative evaluations were carried out by Image J software.

### Statistical analysis

The data were expressed as the means ± SEM from at least three independent experiments. Two-sided *t*-tests were used for comparisons between two groups. A value of P < 0.05 was statistically significant at 95% confidence interval. 1way ANOVA with Dunnett multiple comparisons test was performed for *in vivo* tumor efficacy studies.

## CONCLUSIONS

Given the molecular complexity of cancers, including the extreme heterogeneity in their genetic makeup, their plasticity, innate or acquired resistance to anticancer drugs, there is an increasing need for the development of single-molecule drugs that can effectively and simultaneously impact two or more targets for synergistic anticancer efficacy. We applied a multi-target directed ligand approach to rationally design the hybrid drug KSS19, based on the structures of CA4 and rofecoxib, characterized by unique mechanisms of microtubule disruption and COX-2 inhibition respectively. The structural design of KSS19 also preserved the CA4 nucleus in the cis-configuration and prevented its isomerization to the biologically inactive trans-form. The hybrid drug retained the properties of rofecoxib and CA4 in curtailing the COX-2 activity and microtubule polymerization respectively in cellular and purified *in vitro* systems. Cytotoxicity assays revealed that KSS19 was a potent inhibitor of CRC proliferation both *in vitro* and in xenografts. Most significantly, KSS19 was able to overcome CA4 resistance in colon cancer subtypes exemplified by HT29 cells which express higher levels of the COX-2 enzyme. All assays involving wound heal, migration, invasion, and angiogenesis both *in vitro* and *in vivo* confirmed the excellent antitumor effects than CA4 in HT29 subtype of CRC. KSS19 was also equally potent against other colon cancer cells. In a mouse xenograft model using HT-29 colon cancer cells, KSS19 as a single agent (25 mg/kg) was able to inhibit the tumor growth markedly along with a reduction of intratumoral COX-2 levels. No organ toxicities were evident in xenografted mice indicating the safety profile of the hybrid drug.

Rofecoxib, widely used an NSAID till 14-years ago, was recalled voluntarily by the Merck Pharmaceutical Company due to safety concerns of increased risk of cardiovascular events. However, the data in the literature indicate that rofecoxib carried just a slightly higher risk than other NSAIDS currently in use. Additionally, as a hybrid entity, the KSS19 is likely to have a different pharmacokinetic profile. Also, as a potential anticancer drug, the usage of KSS19 may be short-term. Further, it was less potent in inhibiting Cox enzymes than rofecoxib in tumor cells (Figure 4D). Therefore, KSS19 and its derivatives are likely to be useful in the chemotherapy of colorectal and other cancers.

## Abbreviations

COX-2: cyclooxygenase-2
LPS: lipopolysaccharide
WB: Western blot
DCFDA: 2′
CA4: combretastatin A4
KSS19: the hybrid dug engineered in this study
HUVEC: human umbilical vein endothelial cells
PDB: protein data bank
